# Case Report: Repeated Series of Ketamine Infusions in Patients With Treatment-Resistant Depression: Presentation of Five Cases

**DOI:** 10.3389/fpsyt.2021.705190

**Published:** 2021-12-02

**Authors:** Maria Gałuszko-Wȩgielnik, Adam Włodarczyk, Wiesław Jerzy Cubała, Alina Wilkowska, Natalia Górska, Jakub Słupski

**Affiliations:** Department of Psychiatry, Faculty of Medicine, Medical University of Gdańsk, Gdańsk, Poland

**Keywords:** treatment-resistant depression, ketamine, treatment response, multiple administrations, transformative treatment

## Abstract

**Purpose:** Approximately 30% of patients with major depressive disorder (MDD) are treatment resistant. There is an unquestionable need for new treatment strategies. Subanesthetic doses of intravenous (IV) ketamine have a rapid antidepressant effect in treatment-resistant depression (TRD). This paper describes the efficacy of repeated series of intravenous ketamine infusions as an add-on treatment in five TRD inpatients.

**Methods:** Eligible patients aged 43–63 were given eight ketamine infusions as an add-on treatment for patients with MDD. The subjects have readministered the intervention due to worsening depressive symptoms.

**Results:** Of the five inpatients given ketamine as a series of eight infusions, one underwent three, and four had two treatment series. Four patients achieved remission after first series and three after the second series of ketamine infusions. The adverse reactions were mild and transient with no sequelae.

**Limitations:** Presented case series applies to short-term intervention with IV ketamine as an add-on therapy. The results cannot be generalized to the long-term maintenance treatment nor other ketamine formulations as well as different administration schedules and dosing.

**Conclusions:** This case series showed efficacy and safety of the repeated series of IV ketamine treatment in TRD in MDD and bipolar disorder type I. The subsequent interventions were safe and observed adverse events were mild and transient. Interestingly, the IV ketamine treatment at successive administrations seems to alter the major depression severity of the next affective episode. There is a critical need for further research regarding IV ketamine treatment effectiveness and long-term safety in future studies.

## Introduction

The antidepressant effect of ketamine persists days or weeks beyond treatment, and the assumption may be that the rapid effect will appear, nevertheless, in subsequent ketamine treatment (1).

There is evidence for ketamine use in treatment-resistant depression (TRD) in major depressive disorder (MDD) and bipolar disorder (BD). Current pharmacological treatments for depression prove unsatisfactory efficacy with a proportion of subjects demonstrating TRD. The observation applies both to MDD and BD type I (BD I). Many studies confirmed the antidepressant effect of ketamine used in a single sub-anesthetic intravenous (IV) dose in patients with major depression and TRD patients (2, 3). Subsequent studies confirmed this effect in repeated doses (4, 5), twice-weekly (3, 6, 7), and thrice-weekly (8–10) administration schedules. However, the worsening of depression may occur after infusions are completed, and there is a need for the development of new strategies to maintain the beneficial effects of ketamine treatment. To our knowledge, there are no published data to date on multiple, short-term ketamine administration treatment cycles. This report presents the antidepressant effect of two and three repeated series of IV ketamine in TRD patients with MDD and BD I.

Moreover, ketamine is known to trigger dissociative symptoms with its wide spectrum of sensations, which are one of the major issues of adverse events associated with ketamine intake. These phenomena are to be measured by the Clinician-Administered Dissociative States Scale (CADSS) and Brief Psychiatric Rating Scale (BPRS) are used. That may represent the overall intensity of the dissociative symptomatology (2, 11–13, 30). This case report aims to review safety in course of the treatment of resistant depression in patients with somatic comorbidities.

## Methods

The sample selection methods for this study have been described in detail elsewhere (14). Five inpatients with TRD were administered 0.5 mg/kg ketamine hydrochloride throughout 40 min as an add-on treatment to standard of care. The treatment included two or three series of ketamine infusions (each series included eight infusions performed twice weekly) with safety monitoring.

The psychometric assessment included Montgomery Åsberg Depression Rating Scale structured interview (MADRS-Sigma), Columbia Suicide Severity Rating Scale, and Young Mania Rating Scale completed before infusions and at days 8, 14, 21, and 35. The dissociation and psychomimetic effects scales, the BPRS and the CADSS scales, were completed before every ketamine infusion and 15, 30, 45 min, and 1 h after the infusion, and 1.5 h after the infusion if the two measurements before were >0 points. Qualification procedures and ketamine administration were in line with the APA consensus (15). Psychometric endpoints included rates of response (≥50% reduction from baseline MADRS total score) and rates of remission (MADRS ≤ 12 at day 35).

The cases discussed comprise the naturalistic ketamine registry approved by the bioethical regulatory of the institution (NKBBN/172/2017; 172-674/2019). Patients provided written informed consent for treatment, describing potential risks and limitations as well as reasonable expectations of ketamine treatment for depression.

## Case Series

Patient 1 was a 56-year-old Caucasian male with 9 years of MDD history and six adequately treated MDD episodes. His medical history included hypertension (HTN). The current MDD episode started 7 months before admission. The patient underwent two courses of antidepressants with no effect, and was, therefore, offered ketamine infusions. He achieved remission after eight ketamine infusions. During the first ketamine infusion, we observed a transient blood pressure increase to 180/97 mmHg, but the values returned to normal 123/78 mmHg immediately after the end of ketamine infusion. Ketamine-induced dissociative symptoms were mild and resolved within 0.5 h after each application (CADSS at 15′ = 6 points; BPRS = 1 point). No other adverse events (AEs) appeared during ketamine treatments. The next MDD episode started 5 months after the first one and the patient underwent the second series of eight ketamine infusions with remission lasting the next 6 months. He was admitted again due to a subsequent MDD episode for the third series of infusions, which caused remission sustained for 10-month-long follow-up. During the second and third series, no AEs were observed.

Patient 2 was a 51-year-old Caucasian male diagnosed with MDD at the age of 41 with a history of nine adequately treated MDD episodes. The patient had also been diagnosed with HTN and cerebrovascular incident. The current depression started 13 months before the admission and did not respond to two courses of antidepressants, atypical antipsychotics, and two courses of electroconvulsive therapy (ECT); therefore, he was offered ketamine treatment. After the first series of eight ketamine infusions, he achieved remission lasting 7 months, then MDD appeared. He did not respond to second series of ketamine treatment, but little improvement was observed within the next 12 months. During the first ketamine infusion, his blood pressure increased to 178/95 mmHg and spontaneously dropped to 125/84 mmHg within 30 min. He also had mild dissociative symptoms and dizziness (CADSS at 15′ = 8 points, BPRS = 2 points, at 30′ = 9/1, respectively, and 45′ = 3/0, respectively), which resolved completely within 1 h from the administration. No other AEs were observed. We also did not observe any AEs during the second series of ketamine treatment.

Patient 3 was a 43-year-old Caucasian man diagnosed with BD I 15 years before admission with a history of eight adequately treated manic and 13 adequately treated MDD episodes. He had also been diagnosed with leukemia and dyslipidemia. The current depressive episode, lasting 15 months, failed to respond to one course of antidepressant, augmentation agents, and two ECT courses, and he was offered eight ketamine infusions. After the first series, he achieved remission lasting 9 months. He was admitted again due to further worsening and agreed for the second series of ketamine infusions, which ended with remission sustained for the next 12 months. No manic or hypomanic symptoms during or after ketamine treatment were observed. No AEs during ketamine infusions appeared. CADSS and BPRS scores remained stable, 0 points.

Patient 4 was a 63-year-old Caucasian woman diagnosed with BD I 10 years before admission and had experienced one adequately treated manic and six adequately treated MDD episodes. The current MDD episode started 7 months before and was resistant to one course of antidepressant and one ECT treatment. She received eight ketamine infusions with no response, but a 40% reduction in total MADRS scores; 3 weeks later, due to worsening of depressive symptoms, she received a second series of eight ketamine infusions. However, the reduction in MADRS total scores did not meet response criteria. Dissociative symptoms were also mild and rapidly remitting up to 0.5 h (CADSS at 15′ = 3 points; BPRS = 0 points). No AEs appeared during and between infusions.

Patient 5 was a 60-year-old Caucasian man diagnosed with MDD 15 years before admission with a history of five adequately treated MDD episodes. He had also been diagnosed with obsessive-compulsive disorder, HTN, and psoriatic arthritis. The current depressive episode started 9 months before admission and failed to respond to one course of antidepressant, augmentation agents, and one ECT treatment; therefore, a series of eight ketamine infusions was offered. The patient achieved short-term remission—after 3 weeks, he experienced decline and reported suicidal thoughts. He agreed to the second series of ketamine infusions, which ended with remission lasting for the next 13 months. We observed a rapid reduction of suicidal thoughts after the first ketamine infusion; he also reported mild dissociative symptoms abating within 45 min (CADSS at post-infusions = 5 points; BPRS = 1 point; after 30′ = 2/0, respectively). No AEs during the second series appeared.

## Discussion

**Figure 1:**
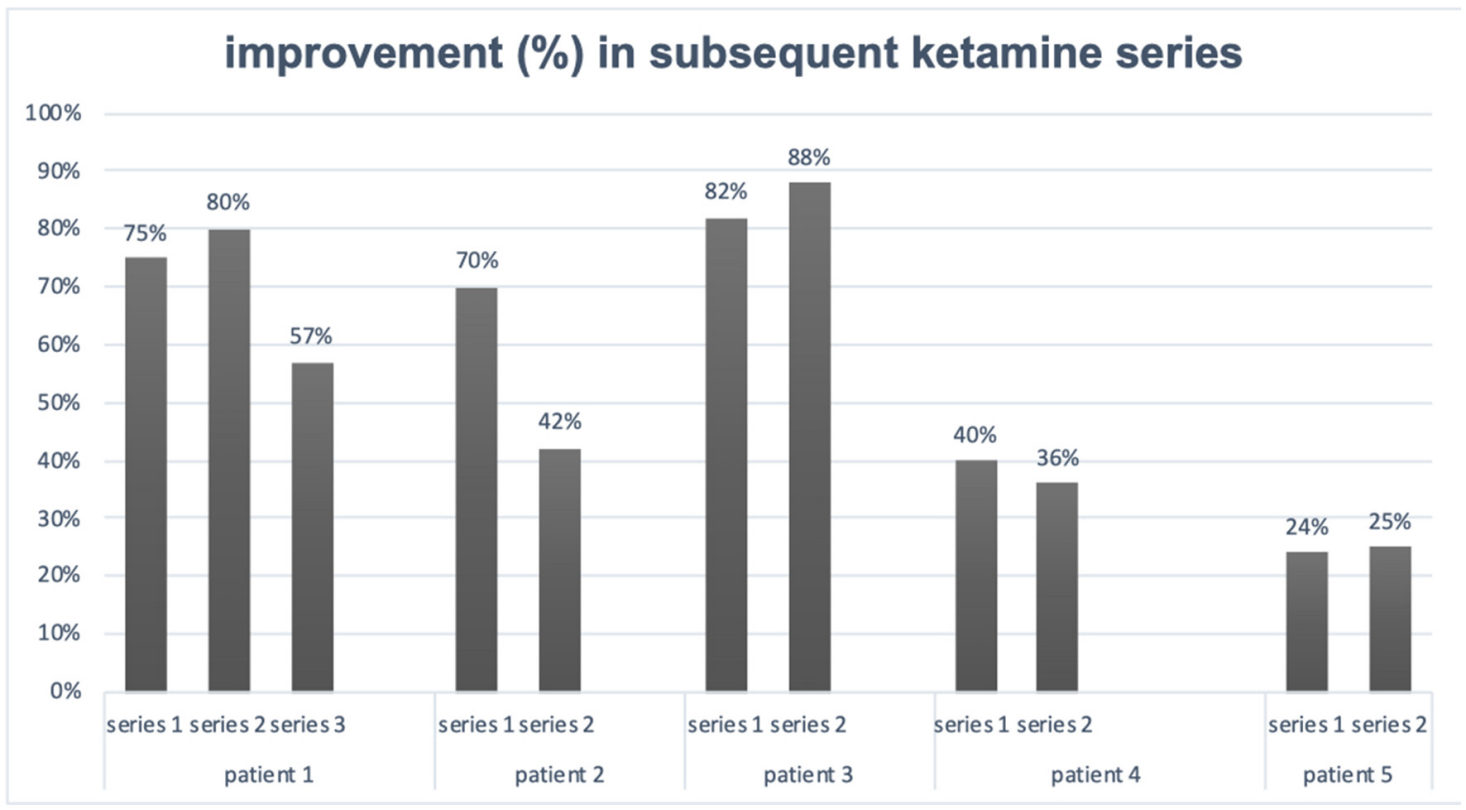
Improvement in subsequent ketamine series.

Here, we report beneficial effects of oral ketamine application in the treatment of patients experiencing TRD and comorbid somatic diseases with regards to general safety, in particular of most common AEs (dissociative and cardiovascular). Although we presented case series and the result must be interpreted with caution, the study shows the restoration of antidepressant response with ketamine after a relapse of depressive symptoms and allowed the direct comparison of response rates for single series and repeated infusions and the general safety profile with regard to dissociative symptomatology. Of the five inpatients given ketamine as a series of eight infusions, one underwent three, and four had two treatment series. Four of five patients achieved remission after the first series and three of five patients met common criteria for remission after the second series of ketamine infusion (Table 1). We noticed that the percentage of change in total MADRS score was lower after the second series than the first, except patient 1. Interestingly, subsequent depressions in patients described were less severe on treatment entry and course (Figure 1). During ketamine treatment, we did not observe any serious AEs, and AEs (dissociative and psychomimetic) that appeared were mild and transient and did not need any rescue medication. Despite multiple comorbidities along with several concomitant medications, the treatment was well-tolerated. The observed effect of ketamine efficacy in subsequent series shall be emphasized as being in line with the esketamine nasal spray studies showing the need for rather continuous than intermittent maintenance administration and with add-on to monoaminergic antidepressants (16).

**Table 1:** Depressive symptoms in the course of ketamine treatment.

		**Pre-treatment MADRS scores:**	**Day 8**	**Day 14**	**Day 21**	**Post-treatment (1 week after final infusion, day 35)**	**Improvement, *n* (%)**
Patient 1	Series 1	37	27	19	10	9	28 (75)
	Series 2	31	19	14	9	6	25 (80)
	Series 3	21	17	11	10	9	12 (57)
Patient 2	Series 1	41	37	32	21	12	29 (70)
	Series 2	33	35	26	19	19	14 (42)
Patient 3	Series 1	40	30	9	6	7	33 (82)
	Series 2	36	8	4	4	4	32 (88)
Patient 4	Series 1	27	19	25	21	16	11 (40)
	Series 2	25	13	10	12	16	9 (36)
Patient 5	Series 1	35	29	19	12	11	24 (68)
	Series 2	16	20	18	9	12	4 (25)

To our knowledge, this is the only study reporting efficacy and safety of repeated series of ketamine infusions. Much of the literature describing the benefits of IV ketamine infusions have been based on single infusions. Single doses have been shown to cause a rapid response and mild side effects, but the antidepressant effect was transient (2, 17). This short-acting effect suggested a need for repeated infusions. Further research confirmed that repeated infusions of ketamine extend the durability of antidepressant efficacy and are more effective than a single dose (4, 10, 18). In the study by Shiroma et al. (10), 14 TRD patients achieved higher response after six ketamine infusions than a single infusion (92 vs. 25%) and the response maintained for 4 weeks, which was also longer compared with a single infusion. Zhan et al. (18) recently reported the response of depression in 59.3% and remission in 40.7% after six ketamine infusions during 12 days in the Chinese population of 86 unipolar and bipolar patients with depression and suicidal ideation. The authors also found the rapid response to suicidal ideation. Phillips et al. (19) conducted a randomized, double-blind crossover comparison of single infusions and continued with 41 TRD participants. The study had three phases: with a single infusion, succeeded by six infusions thrice weekly over 2 weeks, and the maintenance phase with four once-weekly infusions. Bryant et al. (20) reported a series of six geriatric patients treated with acute (5–27) ketamine infusions followed by the long-term maintenance phase (8–22). Five of six patients showed a robust response including further improvement after the acute phase, but they lost the response over time. None of the subjects returned to the baseline level of depression measured by the MADRS, which is in line with our results.

Data on arketamine (another enantiomer of ketamine) are starting to emerge as the open-label pilot trial, including seven subjects with TRD, receiving single intravenous infusion of arketamine (0.5 mg/kg), showed reduction in MADRS scores with almost no dissociative symptoms present. Authors state that arketamine might produce rapid-onset and lasting antidepressant effects in humans with satisfactory safety profile (21). According to Wei et al. (22), it is precarious that NMDAR plays a prevalent role in the antidepressant effects of arketamine. However, at present, the major molecular mechanism by which arketamine exerts its antidepressant actions is unknown.

A systematic review of the safety of ketamine in the treatment of depression, which included 60 studies with 899 patients who had received at least one dose of ketamine, revealed that acute side effects associated with a single dose are common, although generally transient and resolve spontaneously (23). In a study by Wilkinson et al. (7), repeated use (12 or more infusions) was also well-tolerated. In the aforementioned case series by Bryant et al. (20), the authors also did not observe significant side effects although included patients were elderly and had multiple somatic comorbidities; however, the recurrence of addictive behavior appeared in two out of six studied patients. According to the APA, consensus caution is warranted regarding its cardiovascular, cognitive, dissociative, and psychotic effects as well as abuse potential (15). High doses and abuse of ketamine have been associated with potentially serious and possibly persistent toxic effects, including urological, hepatic, but also craving or dependence. To date, these side effects have not been adequately assessed in studies investigating ketamine use in depression. The safety of the long-term, repeated ketamine dosing, as is increasingly used in clinical practice, is therefore uncertain (23).

The use of repeated series of ketamine possibly results in the transformation of the course of the disease (24). Considering that ketamine acts on transcription (25), increases connectivity, neuroplasticity, and cell survival (24), acts on epigenetic mechanisms, and modulates the inflammatory system (26), it may be hypothesized that it exhibits transformative treatment effect modifying the course and the severity of MDD and BP I disorder. This transformation could not only decrease the severity of episodes, reduce suicidality, and extend remissions but also lessen the burden of the stigma associated with mood disorders and become a source of hope to patients and their families (29). The effect may be particularly well-observed across repeated ketamine administration.

The safety and tolerability of dissociative and/or psychotic symptoms induced by antidepressant ketamine and its possible association with a better response to depression reduction were examined. However, currently, the literature does not support the conclusion that dissociation is essential for antidepressant response to ketamine (27). Ketamine is preferred in the treatment of mood disorders for its safety and efficacy, which appear to overlap in several populations, including its use for occasional pain, non-hemodynamic dressings, or sedation (31). This safety report complements the current literature on the safety of ketamine in mood disorders, especially in the TRD-BP population for which limited data are currently available.

Of importance is to acknowledge TRD treatment in line with esketamine nasal spray per FDA-approved label (28), as esketamine nasal spray was proved to be effective in short-term ketamine treatment and, above all, in relapse prevention in TRD-MDD. Although speculative, the case reports presented support long-term relapse prevention in ketamine administration (14). Further studies in a larger population are needed, as well as safety report registries in ketamine/esketamine TRD subjects who discontinued the treatment to observe the relapses and the treatment response pattern to ketamine/esketamine in subsequent depressive episodes.

The presented case series applies to short-term intervention with IV ketamine as an add-on therapy. The results cannot be generalized to the long-term maintenance treatment nor other ketamine formulations (subcutaneous, oral, intranasal, inhalations) as well as different administration schedules and dosing.

## Conclusion

This case series showed the efficacy of the repeated series of IV ketamine treatment in TRD in MDD and BD I. The subsequent interventions were safe and observed AEs were mild and transient. Interestingly, the IV ketamine treatment at successive administrations seems to alter the major depression severity of the next affective episode and impacts the quality of remission with regard to the residual symptoms and suicidality. There is a critical need for further research regarding IV ketamine treatment effectiveness and long-term safety in future studies.

## Data Availability Statement

The raw data supporting the conclusions of this article will be made available by the authors, without undue reservation.

## Ethics Statement

The studies involving human participants were reviewed and approved by Independent Bioethics Committee for Scientific Research at Medical University of Gdańsk, Poland: NKBBN/172-674/2019. https://clinicaltrials.gov/ct2/show/NCT04226963. The patients/participants provided their written informed consent to participate in this study.

## Author Contributions

MG-W and AWł: conceptualization, methodology, and writing—original draft preparation. AWi and WC: validation. AWł and WC: formal analysis. AWi: investigation. AWł: resources, data curation, and writing—review and editing. MG-W and WC: supervision. MG-W, JS, and NG: project administration. WC: funding acquisition. All authors contributed to the article and approved the submitted version.

## Funding

The project was supported by a grant (02-0039/07/221) from the Medical University of Gdańsk.

## Conflict of Interest

MG-W has received research support from Janssen, Servier, Alkermes, KCR, Lilly, Biogen, and Celon. AWł has received research support from Actavis, Eli Lilly, Minerva Neurosciences, Sunovion Pharmaceuticals, KCR, Janssen, Otsuka, Apodemus, Cortexyme, and Acadia. WC has received research support from Actavis, Alkermes, Allergan, Angelini, Auspex, Biogen, Bristol-Myers Squibb, Cephalon, Eli Lilly, Ferrier, Forest Laboratories, Gedeon Richter, GW Pharmaceuticals, Janssen, KCR, Lundbeck, Orion, Otsuka, Sanofi, and Servier; he has served on speakers bureaus for Adamed, Angelini, AstraZeneca, Bristol-Myers Squibb, Celon, GlaxoSmithKline, Janssen, Krka, Lekam, Lundbeck, Novartis, Orion, Pfizer, Polfa Tarchomin, Sanofi, Servier, and Zentiva; and he has served as a consultant for GW Pharmaceuticals, Janssen, KCR, Quintiles, and Roche. AWi has received research support from Janssen, Lilly, Biogen, Celon, Angelini, and Sanofi. JS has received research support from Actavis, Eli Lilly, Minerva, Sunovion, and Celon. NG has received research support from Actavis, Eli Lilly, Minerva, and Sunovion.

## Publisher's Note

All claims expressed in this article are solely those of the authors and do not necessarily represent those of their affiliated organizations, or those of the publisher, the editors and the reviewers. Any product that may be evaluated in this article, or claim that may be made by its manufacturer, is not guaranteed or endorsed by the publisher.

## References

[1] Feifel D Dadiomov D Lee KC. Safety of repeated administration of parenteral ketamine for depression. Pharmaceuticals. (2020) 13:151. 10.3390/ph13070151 32668686 PMC7408561

[2] Berman RM Cappiello A Anand A Oren DA Heninger GR Charney DS. Antidepressant effects of ketamine in depressed patients. Biol Psychiatry. (2000) 47:351–4. 10.1016/S0006-3223(99)00230-9 10686270

[3] Singh JB Fedgchin M Daly EJ De Boer P Cooper K Lim P. A double-blind, randomized, placebo-controlled, dose-frequency study of intravenous ketamine in patients with treatment-resistant depression. Am J Psychiatry. (2016) 173:816–26. 10.1176/appi.ajp.2016.16010037 27056608

[4] Messer M Haller IV Larson P Pattison-Crisostomo J Gessert CE. The use of a series ketamine infusions in two patients with treatment-resistant depression. J Neuropsychiatr Clin Neurosci. (2010) 22:442–4. 10.1176/jnp.2010.22.4.442 21037130

[5] Ghasemi M Kazemi MH Yoosefi A Ghasemi A Paragomi P Amini H. Rapid antidepressant effects of repeated doses of ketamine compared with electroconvulsive therapy in hospitalized patients with major depressive disorder. Psychiatry Res. (2014) 215:355–61. 10.1016/j.psychres.2013.12.008 24374115

[6] Cusin C Ionescu DF Pavone KJ Akeju O Cassano P Taylor N. Ketamine augmentation for outpatients with treatment-resistant depression: preliminary evidence for two-step intravenous dose escalation. Aust N Z J Psychiatry. (2017) 51:55–64. 10.1177/0004867416631828 26893373

[7] Wilkinson ST Katz RB Toprak M Webler R Ostroff RB Sanacora G. Acute and longer-term outcomes using ketamine as a clinical treatment at the Yale Psychiatric Hospital. J Clin Psychiatry. (2018) 79:1–7. 10.4088/JCP.17m11731 30063304 PMC6296748

[8] Murrough JW Perez AM Pillemer S Stern J Parides MK Aan Het Rot M. Rapid and long term antidepressant effects of repeated ketamine infusions in treatment resistant major depression. Biol Psychiatry. (2013) 74:250–6. 10.1016/j.biopsych.2012.06.022 22840761 PMC3725185

[9] Aan het Rot M Collins KA Murrough JW Perez AM Reich DL Charney DS. Safety and efficacy of repeated dose intravenous ketamine for treatment resistant depression. Biol Psychiatry. (2010) 67:139–45. 10.1016/j.biopsych.2009.08.038 19897179

[10] Shiroma PR Johns B Kuskowski M Wels J Thuras P Albott CS. Augmentation of response and remission of serial intravenous subanesthetic ketamine in treatment resistant depression. J Affect Disord. (2014) 155:123–9. 10.1016/j.jad.2013.10.036 24268616

[11] Correia-Melo FS Argolo FC Araújo-de-Freitas L Leal GC Kapczinski F Lacerda AL. Rapid infusion of esketamine for unipolar and bipolar depres- sion: a retrospective chart review. Neuropsychiatr Dis Treat. (2017) 13:1627–32. 10.2147/NDT.S135623 28790825 PMC5488770

[12] McCloud TL Caddy C Jochim J Rendell JM Diamond PR Shuttleworth C. Ketamine and other glutamate receptor modulators for depression in bipolar disorder in adults. Cochr Database Syst Rev. (2015) 29:CD011611. 10.1002/14651858.CD011611.pub2 26415966

[13] Daly EJ Trivedi MH Janik A Li H Zhang Y Li X. Efficacy of esketamine nasal spray plus oral antidepressant treatment for relapse prevention in patients with treatment resistant depression. J Am Med Assoc Psychiatry. (2019) 76:893–903. 10.1001/jamapsychiatry.2019.1189 31166571 PMC6551577

[14] Szarmach J Cubała WJ Włodarczyk A Gałuszko-Wegielnik M. Metabolic risk factors and cardiovascular safety in ketamine use for treatment resistant depression. Neuropsychiatr Dis Treat. (2020) 16:2539–51. 10.2147/NDT.S273287 33154641 PMC7605942

[15] Sanacora G Frye MA McDonald W Mathew SJ Turner MS Schatzberg AF. American Psychiatric Association (APA) council of research task force on novel biomarkers and treatments. A consensus statement on the use of ketamine in the treatment of mood disorders. J Am Med Assoc Psychiatry. (2017) 74:399–405. 10.1001/jamapsychiatry.2017.0080 28249076

[16] Dold M Bartova L Kasper S. Treatment response of add-on esketamine nasal spray in resistant major depression in relation to add-on second-generation antipsychotic treatment. Int J Neuropsychopharmacol. (2020) 29:440–5. 10.1093/ijnp/pyaa034 32570275 PMC7387762

[17] Ballard ED Ionescu DF Vande Voort JL Niciu MJ Richards EM Luckenbaugh DA. Improvement in suicidal ideation after ketamine infusion: relationship to reductions in depression and anxiety. J Psychiatr Res. (2014) 58:161–6. 10.1016/j.jpsychires.2014.07.027 25169854 PMC4163501

[18] Zhan Y Zhang B Zhou Y Zheng W Liu W Wang C. A preliminary study of anti-suicidal efficacy of repeated ketamine infusions in depression with suicidal ideation. J Affect Disord. (2019) 251:205–12. 10.1016/j.jad.2019.03.071 30927581

[19] Phillips JL Norris S Talbot J Birmingham M Hatchard T Ortiz A. Single, repeated, and maintenance ketamine infusions for treatment-resistant depression: a randomized controlled trial. Am J Psychiatry. (2019) 176:401–9. 10.1176/appi.ajp.2018.18070834 30922101

[20] Bryant KA Altinay M Finnegan N Cromer K Dale RM. Effects of repeated intravenous ketamine in treatment-resistant geriatric depression. A case series. J Clin Psychopharmacol. (2019) 39:158–61. 10.1097/JCP.0000000000001006 30742589

[21] Leal GC Bandeira ID Correia-Melo FS Telles M Mello RP Vieira F. Intravenous arketamine for treatment-resistant depression: open-label pilot study. Eur Arch Psychiatry Clin Neurosci. (2021) 271:577–82. 10.1007/s00406-020-01110-5 32078034

[22] Wei Y Chang L Hashimoto K. Molecular mechanisms underlying the antidepressant actions of arketamine: beyond the NMDA receptor. Mol Psychiatry. (2021) 2021:1. 10.1038/s41380-021-01121-1 33963284 PMC8960399

[23] Short B Fong J Galvez V Shelker W Loo CK. Side –effects associated with ketamine use in depression: a systematic review. Lancet Psychiatry. (2018) 5:65–78. 10.1016/S2215-0366(17)30272-9 28757132

[24] Krystal JH Abdallah CG Sanacora G Charney DS Duman RS. Ketamine: a paradigm shift for depression research and treatment. Neuron. (2019) 101:774–8. 10.1016/j.neuron.2019.02.005 30844397 PMC6560624

[25] Harraz MM Tyagi R Cortés P Snyder SH. Antidepressant action of ketamine *via* mTOR is mediated by inhibition of nitrergic Rheb degradation. Mol Psychiatry. (2016) 21:313–9. 10.1038/mp.2015.211 26782056 PMC4830355

[26] Miller AH. Conceptual confluence: kynuramine pathway as a common target for ketamine and the convergence of the inflammation and glutamate hypotheses of depression. Neuropsychopharmacology. (2013) 38:1607–8. 10.1038/npp.2013.140 23857540 PMC3717552

[27] Ballard ED Zarate CA. The role of dissociation in ketamine's antidepressant effects. Nat Commun. (2020) 11:6431. 10.1038/s41467-020-20190-4 33353946 PMC7755908

[28] FDA. Center for Drug Evaluation and Research Approval Letter for Spravato. (2019). Available online at: https://www.accessdata.fda.gov/drugsatfda_docs/nda/2019/211243Orig1s000Approv.pdf (accessed April 15, 2021).

[29] Blackburn TP. Depressive disorders: treatment failures and poor prognosis over the last 50 years. Pharmacol Res Perspect. (2019) 7:3. 10.1002/prp2.472 31065377 PMC6498411

[30] Diazgranados N Ibrahim L Brutsche NE Newberg A Kronstein P Khalife S. A randomized add-on trial of an N-methyl-D-aspartate antagonist in treatment-resistant bipolar depression. Arch Gen Psychiatry. (2015) 67:793–802. 10.1001/archgenpsychiatry.2010.90 20679587 PMC3000408

[31] Page N Nirabhawane V. Intranasal ketamine for the management of incidental pain during wound dressing in cancer patients: a pilot study. Indian J Palliat Care. (2018) 24:58–60. 10.4103/IJPC.IJPC_143_17 29440808 PMC5801631

